# The Use of Sulphuric Acid

**Published:** 1854-12

**Authors:** N. L. North

**Affiliations:** Physician to the Williamsburgh, (L. I.,) Dispensary


ART. III.—*The Use of Sulphuric Acid.* By N. L. NORTH, M. D., Physician to the Williamsburgh, (L. I.,) Dispensary.

Report of cases:

CASE I.—M. L., girl a year and a half old, born at Green Point, L. I. Parents Irish.

July 10. Has had a diarrhœa for three weeks; teething; gums much swollen. Constitutional irritation great.

*Treatment.*—Lanced the gums freely and ordered the following:

℞ Calc. magnes., ʒss.

Pulv. rhei, grs. vi.

Pulv. aromatic, grs. xvij.

Sach. alb., ʒj. M.

Pulv. Dividend, No. 8. Take one three times a day.

Keep the child much in the open air; diet plain but nourishing.

July 11. Patient improved; slept well last night; irritation much less.

Aug. 6. Child greatly emaciated; vomiting very often; dejections too numerous to count, very offensive; rectum prolapsed some three inches; irritability excessive.

Since the last date the child has been better; almost well; so that the mother considered it unnecessary to come back with him. Yet by closer questioning find the intestinal irritation has continued to some extent the whole period, and for several days has been very bad, accompanied with *vomiting* and great debility.

*Treatment.*—Replaced the prolapsed rectum and ordered cold applications; the child to be kept quiet, and the use of castor oil, externally, rubbed over the abdomen thoroughly, and covered with a wide flannel bandage, and the following to be taken internally:

℞ Acid sulph. aromat.,

Mucilage acæ.,

Tr. opii camph., $\overline{aa}$ ℥i. M.

To take a teaspoonful three times a day.

Aug. 7. Patient better. Same treatment continued.

Aug. 8. Still better; vomiting almost entirely ceased; dejections much less frequent, less offensive, and more consistent.

No prolapsus recti. Child desires food. Ordered beef tea, &c., and a continuation of the treatment, except, only to give the acid preparation but twice a day.

Lost sight of the case, after this, for several weeks, when it was found well. The administration of the acid was continued, as last ordered, for some time.

Case II.—W. S., boy two years old. Born and lives at Green Point, L. I. Parents American.

Aug. 30. Has been from home some two months; troubled with diarrhœa much of the time; since his return (some two weeks) has had frequent watery and offensive discharges from the bowels, accompanied with much vomiting, loss of appetite, emaciation, &c. Has been treated with mild laxatives, followed by opiates, astringents, &c., with no improvement. Ordered the following:

℞ Acid sulph. aromat.,

Mucil. acæ.,

Tr. opii camph. āā ℥i. M.

Give a teaspoonful every three hours.

Aug. 31st. Patient decidedly improved. Directed to continue the acid, though only three times a day, and to use castor oil, externally, with the bandage, as in the previous case.

Sept. 2d. Vomiting ceased; dejections only occasional, becoming more natural.

Directed to continue the treatment, withdrawing the acid as the disease gave way.

Sept. 15th. Have occasionally seen the case since the last note, and been pleased to notice the gradual yet uninterrupted return of the healthy functions: the lighting up of the appetite and spirits, increase of strength, &c.

The patient is now well.

The acid preparation was discontinued, and replaced by mild tonics and astringents, and good diet, so soon as the severity of the symptoms had been overcome.

Case III.—F. M., boy, year and a-half old. Born at Green Point. Parents Irish.

Sept. 13 Has had morbid dentition, and intestinal irritation, for months; nothing done. At present teeth through gums, not swollen; but has very offensive, watery, and sometimes bloody discharges from the bowels, with much vomiting and debility. Prescribed at once:

℞ Acid sulph. arom.,

Mucil. acæ.,

Tr. opii camph. $\overline{aa}$ ℥i. M.

To take a teaspoonful three times a day. To return if the child is not better.

Sept. 26. Has not returned, presume the patient well.

CASE IV.—C. G., boy year old. Born in Williamsburgh, L. I. Parents Irish.

Sept. 18. Child brought to the Dispensary for the first time this morning; has been long sick with cholera infantum, irritation from teething, &c.; lies in his mother's arms nearly comatose, noticing nothing of what is passing, unless he is directly addressed, or roughly handled, when he is very irritable.

Prognosis decidedly unfavorable.

*Treatment.*—Scarified the gums and ordered

℞ Acid sulph. aromat., ℥ss.

Mucil. acæ., ℥i.

Tinct. opii campb., ℥ss. M.

Take a teaspoonful three times a day. Rub the abdomen with ol. ricini, and apply flannel bandage. Give *beef broth* to drink.

Sept. 19. Less vomiting; but the mother, misunderstanding directions, gave the acid preparation undiluted, (with water,) and the lips are sore and inflamed.

*Treatment.*—Discontinue the acid and give

℞ Tr. opii camph.,

Tr. Cinchan C., $\overline{aa}$ ℥i.,

Aqua Faut., ℥i. M.

Take a teaspoonful three times a day.

Sept. 20. Worse; give no encouragement at all; will probably die.

Besides the previous difficulty, the change of the weather has produced a good deal of bronchitis, complicating.

*Treatment.*—Ordered of the acid mixture, prescribed the first day, 20 drops every three hours, with castor oil and flannel to the abdomen, and dry friction to the chest occasionally, to be followed by application of warmed flannel.

Beef broth as drink.

Sept. 22. Vomiting seems a little better; has retained a considerable of the broth upon the stomach, which is allowed it often.

Treatment continue as before.

Sept. 23. General appearance about the same, but find the vomiting to have nearly ceased; very thirsty, drinks much of the broth, which is kept down.

Treatment as before.

Sept. 25. Vomiting entirely ceased for the last twenty-four hours. Dejections only *three*, and more natural. General appearance much improved; consider it a signal triumph of the acid treatment, notwithstanding the serious complication of bronchitis.

*Remarks.*—Messrs. Editors: Noticing in the Sept. number of your Journal, an editorial remark upon the subject of cholera, that "the sulphuric acid was a failure in most hands," I have been induced to make the above brief report of four cases of cholera infantum, as a text for a few remarks upon what I consider *the* morbid condition of the system which is under the control of the remedy in question.

For the last two months I have been using it often in the Dispensary, (and occasionally out of it,) with uniform success when given for

Either Markedly Debilitating Diarrhœa,

Or Choleraic Diarrhœa,

Or Cholera Infantum, and from what I have seen of it I deduce the following inferences, viz:

1st. Sulphuric acid in these diseases acts emphatically as a *tonic*, giving *tone* and *action* to the capillary blood vessels.

2d. These diseases consist, (saying nothing of the ultimate or proximate cause of the same,) in a *want of capillary action* and a consequent *passive congestion.*

In the mild forms of diarrhœa from debility, fright, &c., this *want of capillary* action is confined principally to the intestines. In severe cases, it is further extended; and in cholera asphyxia it is extended to almost, or quite the entire system, and excessive *passive* and *internal congestion*, cold skin and extremities, and a straining off of the watery portion of the blood must follow.

3d. The remedy should be given in proportion to the severity of the case, being guided at all times by the *effects*, and not by the quantity given. The reason it was successful in the cases reported above, was because I gave *enough* to control the tendencies of the disease; perhaps one reason "it has proved a failure" in most cases of cholera is, that *enough* has not been given. I might here, perhaps, with propriety mention, the case of a young Englishman who has been a regular attendant of the Dispensary every two or three weeks for some time, having returned from Chagres last spring, worn out with the fever, dysentery, &c. This condition of things followed him up for some time; he was attacked with a severe choleraic diarrhœa, vomiting, &c.; large doses of opium, and calomel and opium, scarcely affected it; I resorted to the following:

℞ Acid sulph. aromat.,

Aqua Faut., āā ℥i. M.

Take a teaspoonful every fifteen minutes till four has been taken, and one every three hours afterward during the day.

The effect was charming; the next day his face was brighter, his appetite sharper, and he supposed himself well.

This was the first of July; some two weeks after he was again similarly attacked, as he has been several times since, produced always by over-exercise, anxiety of feeling, (as he has been in much better circumstances,) or some like cause. But a bold resort to the acid *always* checked it, and without one untoward symptom. Being a *sensible* young man I directed him to take it in such doses as would produce the desired effect, (diluted with pure water,) sometimes he took half drachm doses every fifteen minutes for an hour, and followed with a dose every third hour after, for a day or two; and sometimes he took more, even to drachm doses, with the same intervals of time.

A fourth inference is, that the acid should not be given in any cases of intestinal derangement in which there is asthenic action, *active* congestion, or the *true* inflammatory condition; perhaps its administration in such cases as these is another reason why the remedy has "proved a failure" in arresting intestinal discharges.

5th. I infer that the medicine should be discontinued, or given only in small doses, with lengthened intervals, so soon as the marked morbid condition succumbs to its action, else it may *produce* a disease of itself of a *true inflammatory type.*

